# Predictors of nadir serum creatinine after drainage of bilaterally obstructed kidneys due to different etiologies

**DOI:** 10.1007/s11255-022-03278-2

**Published:** 2022-07-06

**Authors:** Rabea Ahmed Gadelkareem, Ahmed Mahmoud Abdelraouf, Ahmed Mohammed El-Taher, Abdelfattah Ibrahim Ahmed, Nasreldin Mohammed

**Affiliations:** Assiut Urology and Nephrology Hospital, Faculty of Medicine, Assiut University, Elgamaa Street, Assiut, 71515 Egypt

**Keywords:** Bilaterally obstructed kidneys, Double-J stent, Hydronephrosis, Percutaneous nephrostomy, Nadir serum creatinine

## Abstract

**Purpose:**

To identify the predictors of nadir serum creatinine (SCr) after drainage of bilaterally obstructed kidneys (BOKs) by different modes: double-J stent (JJ) versus percutaneous nephrostomy (PCN) and unilateral versus bilateral drainage.

**Methods:**

A prospective non-randomized study was performed on patients with BOKs and raised SCr during December 2019–November 2021. Relevant variables were studied for improvement and non-improvement and for benign and malignant underlying obstructions (BUO and MUO).

**Results:**

This study included 107 patients with BOKs including 68 (63.6%) males and 39 (36.4%) females. After drainage, 86 (80.4%) patients get improved, while 21 (19.6%) patients failed to reach a nadir SCr. Drainage by PCN was significantly higher in MUO, while JJ was significantly higher in BUO (*p* < 0.001). Also, bilateral drainage was a significant predictor of improvement in MUO (*p* = 0.03). In contrast, mode of drainage had no significant effect on improvement in BUO (*p* = 0.84), but bilateral drainage was a significant factor of rapid time to nadir (*p* = 0.02). Univariate analyses revealed no significant effects on the improvement in SCr from the studied variables, except the male gender (*p* = 0.01), old age (*p* < 0.001), MUO (*p* = 0.01), unilateral drainage (*p* < 0.001), and use of PCN for drainage (*p* < 0.001). By multivariate analysis, unilateral drainage (*p* = 0.01) and MUO (*p* < 0.001) were independent predictors of non-improvement in patients with BOKs.

**Conclusions:**

Male gender, old age, MUO, unilateral drainage, and drainage by PCN were significant predictors of non-improvement in SCr after drainage of BOKs. However, unilateral drainage and MUO were the only independent predictors of non-improvement.

## Introduction

The initial relief of obstruction in patients with bilaterally obstructed kidneys (BOKs) is the key point in the proper management and early normalization of renal functions. The underlying causes of obstruction are various between the benign and malignant pathologies [1, 2]. Also, the modalities of drainage have different modes regarding the route and laterality of drainage. All these factors result in variable outcomes, when normalization of the renal function is considered as the primary outcomes [2, 3]. This variability created a state of controversy and non-homogenous research designs that have failed to settle the debates in this subject [1]. On the other hand, the practical nature of this topic with individual experiences and preferences of choosing the mode of management warrants further studying. Regarding the endemism of the commonest underlying pathologies in our region, postrenal acute kidney injury (Po-AKI) due to BOKs puts this topic in a high priority in looking for an evidence-based recommendation for the best mode of drainage in the cases of BOKs [4–6]. We aimed to define the predictors of reaching nadir of the level of post-drainage serum creatinine (SCr) as the most common practical outcome of evaluation of the renal functions.

## Methods

A prospective study was conducted at our hospital from December 2019 to November 2021. This study targeted patients with BOKs. It included patients with age > 18 years, BOKs due to benign or malignant ureteral obstructions (BUO and MUO), high serum creatinine > 2 mg/dl, and grades 1–3 hydronephrosis according the Onen grading system of hydronephrosis [7]. Exclusion criteria were vesicoureteral reflux, bleeding tendency, severe comorbidity preventing intervention, decompensated patients needing urgent dialysis until being compensated by conservation or dialysis, dialysis within 2 weeks from drainage of BOKs, and refusal of participation in the study.

The sample size was calculated using Epi Info version 7.1 for statistical calculation considering a power of the study, 80%, margin of error 10%, confidence level of 90%, and probability value 0.5. A sample size of 97 patients was estimated (the effect size was 0.21). However, considering the percentage of patients with lost to follow-up, we enrolled 110 patients. The number of patients who completed follow-up was 107 patients. This study was conducted in accordance with the principles of the Declaration of Helsinki and its amendments. This study was approved by the local ethical committee at our university, and institutional review board number is 17100860/2019. Also, it was registered in ClinicalTrials: NCT04077008.

In all patients, a full history was taken, including history of loin pain, fever, uremic manifestations as hiccough, vomiting, dyspepsia, anorexia, and urine output, comorbidity, medications, and surgical interventions. Also, systematic physical examination for body temperature, loin tenderness, scar of previous operations, and lower limb edema was done. Laboratory work ups included complete blood count, prothrombin concentration, SCr, blood urea nitrogen, blood gases, random blood sugar, and blood electrolytes. In all cases, imaging studies included ultrasonography, kidney-ureter-bladder radiography, and computed tomography. Magnetic resonance imaging was done only in 4 cases of MUO. Patients were counselled about the available methods of management, and consent for participation in the current study was obtained.

**Fig. 1:**
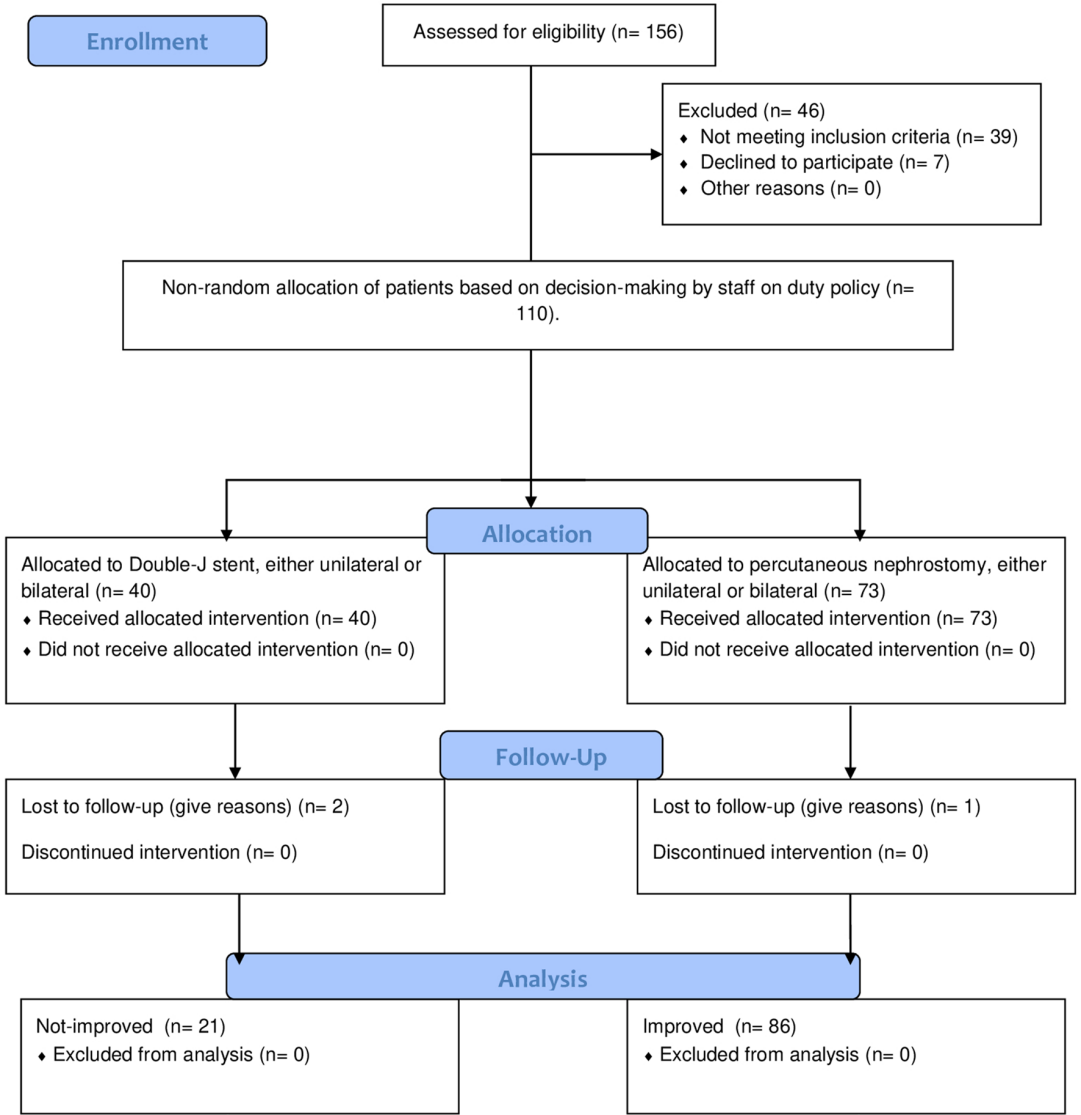
A flowchart of patients underwent drainage of bilaterally obstructed kidneys (BOKs) Sequential steps of the work included assessment for eligibility, counseling, non-random allocation to drainage intervention by percutaneous nephrostomy (PCN) or double-J stent (JJ), follow-up, and data analysis. Patients with infected BOKs or those with malignant ureteral obstruction (MUO) were approached by PCN. Those with BOKs due to benign ureteral obstruction or extraurological malignancies were approached by JJ or PCN. If one modality could not be completed or was refused by the patient, we resorted to the other modality. Three patients were allocated to receive both interventions, one of them on each side. Also, three patients were lost to follow-up. So, the actual numbers in both groups were 69 and 35 patients in the PCN and JJ groups, respectively, plus those with both interventions (3 patients), representing a total of 107 patients

Patients were subjected to drainage of BOKs by Double-J stent (JJ) or percutaneous nephrostomy (PCN). Patients’ flowchart demonstrates their selection, allocation, and management (Fig. 1). Non-random allocation was due to the decision-making policy which was mostly delegated to the staff member on duty or the operator staff member in these emergency cases. The intraoperative and direct postoperative observations were carried out for vital signs, conscious level, and the amount and color of urine. Finally, patients were discharged with instructions of healthcare and follow-up.

The duration of follow-up of patients’ renal function was scheduled over 2 weeks, at 5th, 10th, and 14th day, postoperatively. At each visit, patients were evaluated by physical examination, urine output monitoring, checking PCN patency, SCr, and decompression of kidneys by ultrasonography.

The primary outcome of the study was defined as normalization of SCr or getting two consecutive readings of the lowest SCr value within 2 weeks after drainage of BOKs (nadir SCr level). The secondary outcome was the differences in improvement due to the underlying etiology of BOKs. Accordingly, patients were classified into those who reached a nadir SCr (improved group) and those who failed to reach a nadir SCr (not improved group). Normal SCr was defined as 0.7–1.2 mg/dL, and UOP was defined in different statuses as normal (> 400 ml/day), oliguria (100–400 ml/day), and anuria (< 100 ml/day). Grades of complications were defined according to the modified Clavien classification system [8].

### Statistical analysis

Data were collected and analyzed using SPSS (Statistical Package for the Social Science, version 20, IBM, and Armonk, New York). Quantitative data were expressed as mean ± standard deviation (SD). Nominal data are given as number (*n*) and percentage (%) and compared using Chi-square. Distribution of continuous data was assessed with Shapiro–Wilk test, where normally distributed data were compared using the Student t-test and not normally distributed data were compared using Mann–Whitney U test. In studying these data, patients were classified into 2 groups: improved and not improved according to pervious definitions of values. Predictors of non-improvement were determined by multivariate regression analysis. Level of confidence was kept at 95%, and hence, *P* value was considered significant if < 0.05.

## Results

After exclusion of three patients who lost to follow-up, the current study included 107 patients who had drainage of BOKs. Improved group included 86 (80.4%) patients, while not improved group included 21 (19.6%) patients. The characteristics of patients and the effects of different variables on the primary outcome are demonstrated at different classifications, either according to the improvement in SCr or the underlying etiology (Tables 1, 2, 3, 4, 5, 6,7).

**Table 1:** Demographic and preoperative clinical characteristics of patients in the improved (*n* = 86) and not improved (*n* = 21) groups (total *n* = 107)

Characteristics	Improved (*n* = 86)	Not improved (*n* = 21)	*P* value
Age (years)	53.95 ± 13.18	67.23 ± 10.86	< 0.001
Gender			
Male	50 (58.1%)	18 (85.7%)	0.01
Female	36 (41.9%)	3 (14.3%)	
BMI (kg/m^2^)	25.53 ± 5.55	24.25 ± 5.31	0.34
Comorbidity			
Diabetes mellitus	21 (24.4%)	3 (14.3%)	0.24
Hypertension	17 (19.8%)	5 (23.8%)	0.44
Cardiac diseases	6 (7%)	2 (9.5%)	0.49
Pulmonary diseases	0	1 (4.8%)	0.19
Smoking	12 (14%)	5 (23.8%)	0.21
Previous surgical interventions			
Open kidney surgery	1 (1.2%)	0	0.80
PCN	2 (2.3%)	1 (4.8%)	0.48
Renal SWL	3 (3.5%)	0	0.51
Open urethral surgery	3 (3.5%)	0	0.51
URS	1 (1.2%)	0	0.80
Double-J stent	3 (3.5%)	0	0.51
Loin pain			
Unilateral	5 (5.8%)	0	0.21
Bilateral	41 (47.7%)	14 (66.7%)	
None	40 (46.5%)	7 (33.3%)	
Urine output at presentation			
Normal	34 (39.5%)	12 (57.1%)	0.20
Oliguria	28 (32.6%)	6 (28.6%)	
Anuria	24 (27.9%)	3 (14.3%)	
Uremic manifestations			
Hiccough	8 (9.3%)	2 (9.5%)	0.62
Vomiting	12 (14%)	1 (4.8%)	0.22
None	74 (87.1%)	19 (90.5%)	0.50
Temperature (°C)	37.19 ± 0.51	37.36 ± 0.61	0.18
Loin tenderness			
Unilateral	3 (3.5%)	1 (4.8%)	0.41
Bilateral	8 (9.3%)	4 (19%)	
None	75 (87.2%)	16 (76.2%)	
Pre-drainage dialysis sessions			
None	75 (87.2%)	19 (90.5%)	0.68
Once	3 (3.5%)	0	
Twice and more	8 (9.3%)	2 (9.5%)	
Underlying obstruction nature			
Malignant ureteral obstruction	36 (41.9%)	18 (85.7%)	< 0.001
Benign ureteral obstruction	50 (58.1%)	3 (14.3%)	

*BMI* body mass index, *PCN* percutaneous nephrostomy, *SWL* extracorporeal shockwave lithotripsy, *URS*: ureteroscopy

**Table 2:** Effects of the preoperative laboratory and imaging characteristics on the primary outcome in all patients

Characteristics	Improved (*n* = 86) Mean ± SD	Not improved (*n* = 21) Mean ± SD	*P* value
Parenchymal thickness (cm^3^)			
Right kidney	13.17 ± 2.44	12.86 ± 2.45	0.59
Left kidney	12.73 ± 2.41	13.23 ± 2.34	0.38
Renal length (cm)			
Right kidney	11.91 ± 1.52	11.02 ± 1.29	0.15
Left kidney	11.74 ± 1.62	11.48 ± 1.37	0.50
Serum creatinine (mg/dl)	5.90 ± 3.07	7.06 ± 3.59	0.13
Random blood sugar (mg/dl)	127.45 ± 49.60	134.81 ± 88.42	0.61
pH	7.35 ± 0.07	7.35 ± 0.08	0.91
CO_2_	25.35 ± 6.19	22.80 ± 4.91	0.08
Acid–base deficit (mmol/l)	-8.48 ± 5.55	-9.79 ± 5.34	0.33
HCO_3_^+^	15.44 ± 4.25	14.71 ± 3.32	0.46
Leucocytes (cells × 10^3^/ul)	8.23 ± 2.96	9.40 ± 3.99	0.13
Hemoglobin (gm/dl)	10.98 ± 1.61	10.73 ± 1.35	0.49
Pyuria (cells/HPF)^a^	42.44 ± 35.14	42.09 ± 35.99	0.96

^a^Performed only for patients with urine output allowed sampling

*CO*_*2*_ carbon dioxide, *HCO*_*3*_^+^ bicarbonate, *pH* potential hydrogen describing the acidity or basicity of blood

**Table 3:** Effect of the nature of the underlying obstruction on the primary outcome in all patients

Underlying obstruction	Improved (*n* = 86)	Not improved (*n* = 21)	*P* value
Benign ureteral obstruction			0.01
Urolithiasis	39 (45.3%)	4 (19.1%)	
Ureteral stricture	6 (6.9%)	0	
Iatrogenic ureteric injury	2 (2.3%)	0	
Retroperitoneal fibrosis	2 (2.3%)	0	
Malignant ureteral obstruction			
Urinary bladder cancer	17 (19.8%)	13 (61.9%)	
Cancer prostate	3 (3.4%)	2 (14.3%)	
Cervical cancer	5 (5.8%)	1 (4.8%)	
Cancer colon	5 (5.8%)	1 (4.8%)	
Cancer rectum	5 (5.8%)	0	
Lymphoma	2 (2.3%)	0

**Table 4:** Effect of the mode of drainage on the primary outcome in all patients

Variables	Improved (*n* = 86)	Not improved (*n* = 21)	*P* value
Mode of drainage			< 0.001
Unilateral PCN	28 (32.5%)	17 (81%)	
Bilateral PCN	22 (25.6%)	2 (9.5%)	
Unilateral JJ	9 (10.5%)	0	
Bilateral JJ	24 (27.9%)	2 (9.5%)	
PCN/JJ	3 (3.5%)	0	
Laterality of drainage			< 0.001
Unilateral drainage	37 (43%)	17 (81%)	
Bilateral drainage	49 (57%)	4 (19%)	
Type of catheter^a^			< 0.001
JJ	33 (39.8%)	2 (9.5%)	
PCN	50 (60.2%)	19 (90.5%)	

*JJ* double-J stent, *PCN* percutaneous nephrostomy

^a^The three patients who underwent double-J on one side and percutaneous nephrostomy (PCN) on the other side weren’t included in comparison of type of catheter

**Table 5:** Differences in demographic and clinical characteristics of groups of patients with benign and malignant ureteral obstructions

Variables	Benign ureteral obstruction (*n* = 53)	Malignant ureteral obstruction (*n* = 54)	*P* value
	Mean ± SD/frequency (percentage)		
Age (year)	53 ± 11.9	60 ± 14.7	< 0.001
Gender			
Male	31 (58.5%)	37 (68.5%)	0.381
Female	22 (41.5%)	17 (31.5%)	
Height (m)	1.66 ± 0.06	1.64 ± 0.07	0.120
Weight (kg)	72.2 ± 16.9	66.9 ± 19	0.066
BMI (kg/m^2^)	26 ± 5.3	24.5 ± 5.4	0.091
Comorbidities^a^			
Diabetes mellitus	10 (18.9%)	14 (25.9%)	0.458
Hypertension	10 (18.9%)	12 (22.2%)	
Cardiac diseases	3 (5.7%)	5 (9.3%)	
Pulmonary diseases	1 (1.9%)	0 (0%)	
None	29 (54.7%)	23 (42.6%)	
Previous surgical interventions	8 (15.1%)	6 (11.1%)	0.932
Smoking	9 (17%)	8 (14.8%)	0.775
Pain laterality			
Right	0 (0%)	3 (5.6%)	0.067
Left	1 (1.9%)	1 (1.9%)	
Bilateral	34 (64.1%)	21 (38.9%)	
None	18 (34%)	29 (53.7%)	
Temperature (°C)	37.2 ± 0.5	37.3 ± 0.6	0.273
UOP at presentation			
Normal	13 (24.5%)	33 (61.1%)	< 0.001
Oliguria	21 (39.6%)	13 (24.1%)	
Anuria	19 (35.9%)	8 (14.8%)	
Loin tenderness			
Unilateral	1 (1.9%)	3 (5.6%)	0.753
Bilateral	5 (9.4%)	7 (13%)	
None	47 (88.7%)	44 (81.5%)	
Pre-drainage dialysis			
Yes	5 (9.4%)	8 (14.8%)	0.852
None	48 (90.6%)	46 (85.2%)	
Uremic symptoms			
Yes	10 (13.2%)	13 (24.1%)	0.668
None	43 (81.1%)	44 (81.5%)	
Mean parenchymal thickness of drained unit(s) (mm)	13.4 ± 2.3	12.8 ± 2.3	0.151
SCr at presentation (mg/dl)	6.2 ± 2.9	6.1 ± 3.5	0.501
eGFR at presentation (ml/min/1.73 m^2^)	12.8 ± 9.5	13.1 ± 8.1	0.510
Random blood sugar (mg/dl)	128.4 ± 53.2	129.5 ± 64.3	0.519
pH	7.35 ± 0.07	7.36 ± 0.08	0.615
PCO_2_ (mmHg)	25.7 ± 5.4	24 ± 6.5	0.125
Acid–base deficit (mmol/l)	− 9.1 ± 5.1	− 8.4 ± 5.9	0.549
HCO_3_ (mmol/l)	15.1 ± 4.1	15.5 ± 4.1	0.570
Leucocytes (Cells × 10^3^/ul)	8.7 ± 3.3	8.2 ± 3.1	0.408
Hemoglobin (gm/dl)	10.9 ± 1.7	11 ± 1.4	0.861
Mode of drainage			
Unilateral PCN	12 (22.6%)	33 (61.1%)	< 0.001
Bilateral PCN	6 (11.3%)	18 (33.3%)	
Unilateral JJ	9 (17%)	0 (0%)	
Bilateral JJ stent	25 (47.2%)	1 (1.9%)	
PCN/JJ stent	1 (1.9%)	2 (3.7%)	
Post-drainage mean UOP (ml)	2230 ± 940	2050 ± 650	0.554
Post-drainage mean weight (kg)	71.6 ± 16.8	66.5 ± 18.7	0.070
Post-drainage kidney function			
SCr at 5th day (mg/dl)	2.8 ± 2.1	3.3 ± 2.3	0.172
SCr at 10th day (mg/dl)	2 ± 1.8	2.6 ± 2	0.08
SCr at 14th day (mg/dl)	1.3 ± 1.3	1.7 ± 1.1	0.007
eGFR at lowest SCr level (ml/min/1.73 m^2^)	85 ± 41.2	63.4 ± 38.8	0.010

*BMI* body mass index, *CI* confidence interval, *eGFR* estimated glomerular filtration rate, *HCO3* + bicarbonate, *JJ* double-J stent, *PCN* percutaneous nephrostomy, *PCO2* blood carbon dioxide, *pH* potential hydrogen describing the acidity or basicity of blood, *SCr* serum creatinine, *SD* standard deviation, *UOP* urine output

^a^In the group of malignant ureteral obstruction, only 6 patients had a history of chemotherapy regimen

**Table 6:** Effect of modes of drainage per the underlying obstruction on the primary outcome

Modes of drainage per category of underlying ureteral obstruction	Frequency per outcome groups		*P* value
*Malignant ureteral obstruction*	Improved (*n* = 36)	Not improved (*n* = 18)	
Mode of drainage			0.03
Unilateral PCN	17 (47.2%)	16 (88.9%)	
Bilateral PCN	16 (44.4%)	2 (11.1%)	
Bilateral JJ	1 (2.8%)	0	
PCN/ JJ	2 (5.6%)	0	
Laterality of drainage			0.03
Bilateral drainage	19 (52.7%)	2 (11.1%)	
Unilateral drainage	17 (47%)	16 (88.9%)	
*Benign ureteral obstruction*	Improved (*n* = 50)	Not improved (*n* = 3)	
Mode of drainage			0.84
Unilateral PCN	11 (22%)	1 (33.3%)	
Bilateral PCN	6 (12%)	0	
Unilateral JJ	9 (18%)	0	
Bilateral JJ	23 (46%)	2 (66.7%)	
PCN/JJ	1 (2%)	0	
Laterality of drainage			0.49
Bilateral drainage	30 (60%)	2 (66.7%)	
Unilateral drainage	20 (40%)	1 (33.3%)	

*JJ* double-J stent, *PCN* percutaneous nephrostomy

**Table 7:** Multivariate regression analysis of the predictors of non-improvement in serum creatinine after drainage in patients with bilaterally obstructed kidneys

Variables	Odds ratio	95% CI	*P* value
Age > 60 years	3.19	0.82–12.43	0.09
Male gender	4.30	0.92–17.77	0.06
Malignant ureteral obstruction	11.23	1.62–22.87	< 0.001
Double-J stent	0.27	0.25–2.97	0.28
Unilateral drainage	5.88	1.53–11.76	0.01

*P* value was significant if < 0.05

*CI* confidence interval

The current study included 68 (63.6%) males and 39 (36.4%) females. Totally, the mean age was 56.56 ± 13.8 years and the mean BMI was 25.28 ± 5.5 kg/m^2^. The characteristics of the patients relative to their grouping into BUO and MUO are summarized in Table 5.

BUO was the underlying cause in 53 (49.5%) patients, including urolithiasis in 43 (40%), ureteral stricture in 6 (5.6%), iatrogenic ureteral injury in 2 (1.9%), and retroperitoneal fibrosis in 2 (1.9%) patients. However, MUO occurred in 54 (50.5%) patients, including urinary bladder cancer in 30 (28%), prostate cancer in 5 (4.7%), cervical cancer in 6 (5.6%), colorectal cancer in 11 (10.3%), and lymphoma in 2 (1.9%) patients.

The total mean SCr at presentation was 6.13 ± 3.2 mg/dl. There were no statistically significant differences between both groups in the preoperative laboratory and imaging variables (Table 2). The underlying pathology of ureteral obstruction was differentiated into BUO and MUO with subcategories. They showed significant differences in their effects on the improvement in SCr level (Table 3).

Regarding the mode of drainage of BOKs (Tables 4, 5, 6), 57% of patients in the improved group underwent bilateral drainage, while the majority (81%) of patients in the not improved group underwent unilateral drainage. Also, with the exception of 2 patients, all those patients who did not improve had PCN (Table 4). The majority of the patients with BUO were managed by JJ, while most of patients with MUO were managed by PCN (*P* < 0.001) (Tables 5 and 6).

**Fig. 2:**
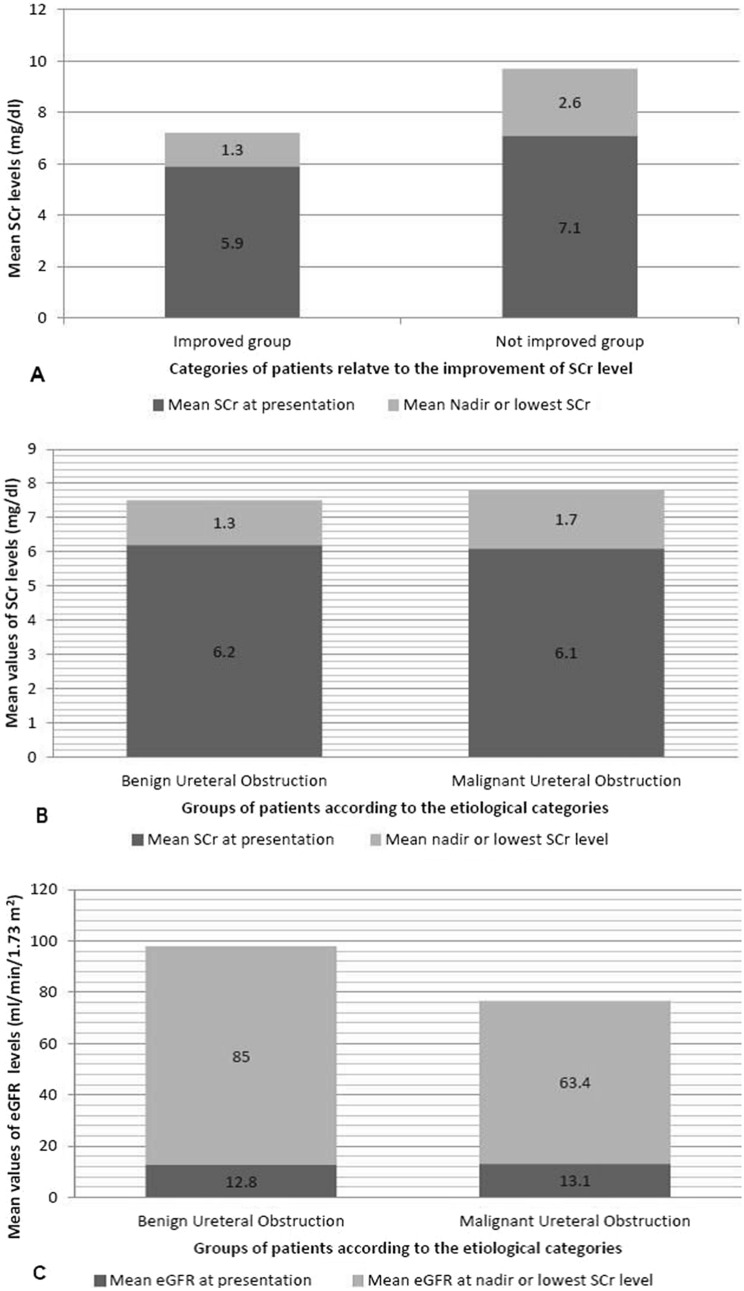
Graphical representations of the pre- and post-drainage values of renal functions. It shows the differences between the mean serum creatinine (SCr) levels (**A** and **B**) and the estimated glomerular filtration rate (eGFR) (**C**), at presentation and at nadir SCr levels for different groups of patients. Between the improved and not improved groups, the values of Cohen’s *d*, Glass's delta, and Hedges' *g* tests for mean SCr at presentation/nadir levels were 0.36/1.49, 0.39/1.36, and 0.37/1.59, respectively. However, between the benign and malignant ureteral obstruction (BUO and MUO) groups, the effect size values of Cohen’s *d*, Glass's delta, and Hedges' *g* for mean SCr at presentation/nadir levels were 0.03/0.33, 0.03/0.31, and 0.03/0.33, respectively. Also, the corresponding values for mean eGFR in BUO and MUO groups were 0.03/0.54, 0.03/0.52, and 0.03/0.54, respectively

The mean SCr at 5th, 10th, and 14th day of follow-up was 3 ± 2.2, 1.8 ± 1.4, and 1.49 ± 1.19. The mean nadir SCr in the improvement group was 1.13 ± 0.88 mg/dl. The mean SCr in patients with normalized SCr and in patients who reached an abnormal nadir SCr was 0.7 ± 0.3 mg/dl and 2.9 ± 1.2 mg/dl. The differences between the mean SCr at presentation and at the lowest post-drainage level were compared between both groups. Also, these values were compared between BUO and MUO groups (Fig. 2).

The mean of the total time-to-nadir SCr was insignificantly different (*P* = 0.17) between the unilateral (9.11 ± 4.01 days) and bilateral (7.88 ± 4.11 days) drainage. In MUO, the mean time to nadir was also insignificantly different (*P* = 0.18) between the bilateral (8.06 ± 3.91 days) and unilateral (8.65 ± 4.74 days) drainage. In BUO, however, the mean time-to-nadir SCr was significantly longer (*P* = 0.02) in the unilateral drainage (10 ± 3.97 days) than it in the bilateral drainage (7.37 ± 3.87 days).

By multivariate analysis, the independent predictors of non-improvement were only MUO (*p* < 0.001) and unilateral drainage (*p* = 0.01) (Table 7).

Regarding the complications, there were 2 cases that were converted to PCN after failure of JJ insertion (Grade 1). Also, 1 patient had failed PCN placement and converted to JJ (Grade 3a). Postoperative hematuria occurred in 5 (14%) patients of JJ group and 9 (12.5%) patients in PCN group (Grade 2). All those cases were treated conservatively without blood transfusion. At follow-up visits, 17 patients complained of irritative lower urinary tract symptoms after JJ placement (Grade 2). PCN slippage occurred only in one patient, where it was repositioned within 3 h.

## Discussion

BOKs is a classic form of acute kidney injury that could be cured by resolution of the obstructing factor. Drainage can be achieved by placement of PCN or JJ until stabilization of the renal functions and arrangement for the primary treatment. Stabilization of renal function is usually monitored by the decrease in the level of SCr. Many factors may predict the recoverability of renal function, including the tool and laterality of drainage. These predictors have been variably studied without reaching a consensus on the optimal strategy of drainage of BOKs [1, 9]. Here, we conducted this prospective study considering the nadir SCr as the primary outcome.

Overall, the most common cause of obstruction in the current study was urolithiasis (40%). This was consistent with the data reported in most of the literature, regarding urolithiasis as the most common cause of ureteric obstruction in Po-AKI. This includes studies from our locality that reported urolithiasis as the main cause of obstruction [1, 5, 6, 10].

In our study, urological malignancies represented 65% of MUO, where bladder cancer was the most common cause (55.6%). These results were similar to those in the studies by Cordeiro et al. [11] and Haas et al. [12]. The latter conducted the largest study in the literature on 238,500 cases of MUO [12]. They reported bladder cancer (23%) and prostate cancer (17.9%) as the most common causes of MUO. In contrast, Kanou et al. [13] and Aekgawong et al. [14] found that the extra-urological malignancies such as cervical cancer are more common than the other variants of obstructing malignancies. However, many studies reported that genitourinary malignancies represent more than 25% of MUO causes [15–17].

On average, the preoperative SCr was 6.13 ± 3.2 mg/dl. It had no statistically significant effect the improvement in SCr which was similar to the results [18–20]. However, it was different from the previous results found that the low SCr at presentation was a statistically significant predictor for recoverability of renal functions in patients with Po-AKI [5, 14, 21].

BUO may have criteria different from MUO. They may allow passing a ureteral stent through the ureter. Also, the curable nature may promote the urologist to try all the efforts to keep the normal passage. Moreover, patients with BUO such as that due to urolithiasis usually have no systematic dysfunctions. Accordingly, they have intact physiological and metabolic compensatory responses or mechanisms. The latter processes are usually reflected on the patient’s physical conditions, functional performance, and they can competently compensate for the pathophysiological sequels of AKI after drainage. These functional competences may help making a decision of invasive interventions such as JJ placement [1, 6, 18, 19]. This is in contrast with patients with MUO who have bad general performance and short life expectancy due to the advanced malignancy [20, 22].

MUO has two main mechanisms for development of AKI. Firstly, it causes mechanical obstruction of the ureters like any other cause hindering the urine flow. Secondly, it has its own metabolic insults that affect the whole body environment and the concomitant burden of medications such as chemotherapy [2].

According to the current study, bilateral drainage of BOKs has a statistically significant effect on the recovery of renal function. It could significantly increase the possibility of renal function recovery in MUO. Also, it could significantly make the time-to-nadir SCr shorter in BUO. Similarly, many studies reported a significant effect of bilateral drainage on the recovery of renal functions [14, 23].

Although urologists meet with the cases of BOKs mandating drainage, there are no guidelines addressing the recommendation to the preferences of laterality of drainage [2, 24]. In our study, nadir SCr was 1.13 ± 0.88 mg/dl with a significantly longer time to nadir in cases of unilateral drainage than that in bilateral drainage of BOKs. Also, we found that time-to-nadir SCr in patients with BUO and bilateral drainage was significantly lower than that with unilateral drainage (*P* < 0.02). In contrast, some studies found no significant effect of drainage laterality on reaching a nadir SCr and reported a time-to-nadir SCr of 7.7–10 days [18, 24]. However, most of these studies did not exclude patients with severe hydronephrosis. It is important to exclude patients with non-functioning or poorly functioning kidneys, when laterality is compared.

In our study, MUO was an independent predictive factor for non-improvement after successful drainage. This was consistent with many studies in the literature [11, 22, 25]. Moreover, other studies reported that improved patients with MUO would need longer time-to-improve up to > 15 days [16, 26], which was comparable to our results.

The current results showed that the rate of improvement in SCr after drainage by JJ was significantly higher than it after drainage by PCN. This finding was inconsistent with many previous studies that found no difference between both of these methods of drainage [5, 27]. This could be attributed to the preference to use PCN in cases of MUO in our hospital, where this association might be the reason that why it lost the statistical significance after incorporation in multivariate analysis. On the other hand, JJ may have lower success rates and higher rates of complications in MUO [28].

The previous studies reported many independent factors of recovery of renal functions after drainage of BOKs such as symptom duration of < 25 days, the patient’s age, and low platelet count and serum albumin at presentation [1, 2, 5, 14, 19]. In parallel, MUO and unilateral drainage were independent predictors in our results. However, patients in these studies were different from ours; they had Po-AKI in patients with only-functioning kidneys due to BUO only such as urolithiasis or MUO only [2, 5, 14, 19, 29].

The limitations of the current study included the non-random allocation of patients in receiving the mode of drainage. Hence, the sufficiency of the current results for drawing a very solid evidence could be criticized due to a proposed bias in patient selection for a certain mode of drainage by the operator. Also, the scope of this study did not withstand studying quality of life, patients’ survival [30], and whether the effect of normalization of SCr enabled those patients to catch earlier dates for correction of the underlying causes. Furthermore, reliable recording of the daily changes in UOP and body weight was not amenable after discharging the patients from the hospital within 1–2 days. Besides, generalizability of the results might be limited as the study was single-center one. However, the cumulative evidence in the literature may alleviate these limitations.

## Conclusions

BOKs had various underlying etiologies, including urolithiasis as the most common benign cause and bladder cancer as the most common malignant cause. Old males were more affected rather than females. There were numerous modes of drainage of BOKs, regarding the type of catheter and the laterality of drainage. The univariate analyses showed that the predictive factors for non-improvement in renal function represented by SCr level after drainage of BOKs included the male gender, old age, MUO, unilateral drainage, and use of PCN for drainage. By multivariate analysis, however, unilateral drainage and MUO were the only independent predictors of non-improvement after drainage of BOKs. Bilateral drainage was superior to unilateral drainage in the reduction in SCr level to a nadir. Time-to-nadir SCr was significantly shorter in BUO than in MUO.
